# Supplementary material for: The Controversial Effect of Antibiotics on Methicillin-Sensitive S. aureus: A Comparative In Vitro Study
Source: Int J Mol Sci. 2023 Nov 14;24(22):16308. doi: 10.3390/ijms242216308 (PMC10671744; doi:10.3390/ijms242216308)
Supplement: Supplementary file 1 [file ijms-24-16308-s001.zip › ijms-2672359-SI.pdf]

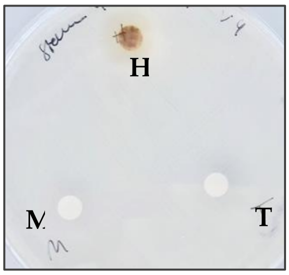

Figure S1. Auxotrophism test of SCV derived from the strain 6850 with hemin (H), menadione (M) and thymidine (T) after 3 days of incubation.
